# The Impact of Prenatal Alcohol Exposure on the Autonomic Nervous System and Cardiovascular System in Rats in a Sex-Specific Manner

**DOI:** 10.3390/pediatric16020024

**Published:** 2024-04-09

**Authors:** Michał Jurczyk, Magdalena Król, Aleksandra Midro, Katarzyna Dyląg, Magdalena Kurnik-Łucka, Kamil Skowron, Krzysztof Gil

**Affiliations:** 1 Department of Pathophysiology, Faculty of Medicine, Jagiellonian University Medical College, Czysta 18, 31-121 Krakow, Poland; 2 St. Louis Children Hospital, Strzelecka 2, 31-503 Krakow, Poland

**Keywords:** fetal alcohol spectrum disorders, prenatal alcohol exposure, heart rate variability

## Abstract

Background: Fetal Alcohol Spectrum Disorder (FASD) is a consequence of prenatal alcohol exposure (PAE) associated with a range of effects, including dysmorphic features, prenatal and/or postnatal growth problems, and neurodevelopmental difficulties. Despite advances in treatment methods, there are still gaps in knowledge that highlight the need for further research. The study investigates the effect of PAE on the autonomic system, including sex differences that may aid in early FASD diagnosis, which is essential for effective interventions. Methods: During gestational days 5 to 20, five pregnant female Wistar rats were orally administered either glucose or ethanol. After 22 days, 26 offspring were born and kept with their mothers for 21 days before being isolated. Electrocardiographic recordings were taken on the 29th and 64th day. Heart rate variability (HRV) parameters were collected, including heart rate (HR), standard deviation (SD), standard deviation of normal-to-normal intervals (SDNN), and the root mean square of successive differences between normal heartbeats (RMSSD). Additionally, a biochemical analysis of basic serum parameters was performed on day 68 of the study. Results: The study found that PAE had a significant impact on HRV. While electrolyte homeostasis remained mostly unaffected, sex differences were observed across various parameters in both control and PAE groups, highlighting the sex-specific effects of PAE. Specifically, the PAE group had lower mean heart rates, particularly among females, and higher SDNN and RMSSD values. Additionally, there was a shift towards parasympathetic activity and a reduction in heart rate entropy in the PAE group. Biochemical changes induced by PAE were also observed, including elevated levels of alanine transaminase (ALT) and aspartate aminotransferase (AST), especially in males, increased creatinine concentration in females, and alterations in lipid metabolism. Conclusions: PAE negatively affects the development of the autonomic nervous system, resulting in decreased heart rate and altered sympathetic activity. PAE also induces cardiovascular abnormalities with sex-specific effects, highlighting a relationship between PAE consequences and sex. Elevated liver enzymes in the PAE group may indicate direct toxic effects, while increased creatinine levels, particularly in females, may suggest an influence on nephrogenesis and vascular function. The reduced potassium content may be linked to hypothalamus–pituitary–adrenal axis overactivity.

## 1. Introduction

Fetal Alcohol Spectrum Disorders (FASDs) represent a significant challenge in contemporary medicine. This condition was initially described in 1973 in two articles and referred to as “fetal alcohol syndrome” (FAS) [1,2]. These articles highlighted a range of congenital malformations caused by prenatal alcohol exposure (PAE). Since then, the general understanding of FASD has grown to represent a spectrum of physical, neurodevelopmental, behavioral, and cognitive impairments with varying degrees of severity. This expanded knowledge has led to improved diagnostic criteria and intervention strategies to support affected individuals and their families [3].

Throughout the years, multiple animal models have been developed to explore the effects of PAE [4]. Rodents, including mice and rats, are frequently used due to their genetic similarities to humans. Their short gestation period and well-known reproductive biology make them valuable subjects for investigating the neurobiological effects of PAE [5]. Administration of alcohol throughout the early postnatal period, which reflects the third trimester in humans, has garnered significant interest due to its enduring influence on neurogenesis [6]. In fact, several specific rat models of PAE have been previously developed, each with its own advantages and disadvantages [7]. The liquid diet approach, when combined with alcohol, enables a low-to-moderate level of ethanol exposure. Yet, its major drawback is the inherent difficulty to accurately select the desired quantity of ethanol [8]. The voluntary drinking model can be established by training female rats to spontaneously consume a solution of ethanol sweetened with saccharin. This model facilitates low-to-moderate exposure to alcohol but without precise dose control. On the other hand, intragastric models involve the direct administration of a specified volume of ethanol. However, these models are accompanied by additional procedures that have the potential to cause injury or induce stress [9]. The injection models are based on subcutaneous or intraperitoneal injections of ethanol, which allow for an accurate dosage and a fast elevation in blood ethanol levels while minimizing stress generated by manipulation. Nevertheless, this route of administration bears little resemblance to the ingestion of ethanol in humans [10]. The inhalation mode of administration is the least commonly used in animal models of PAE. Accordingly, pregnant dams or the dam and her neonates are placed in an inhalation chamber filled with ethanol vapor for a defined duration [11]. This method causes a rapid and reliable increase in blood alcohol concentration without any stress of oral administration and prolonged handling. Yet, this delivery method is quite distinct from the intake of ethanol by people [12]. PAE is commonly associated with a decrease in the overall volume of the brain, which may be noticed both at birth and throughout life. Individuals with a history of PAE may have a decrease in both gray and white matter in the cerebrum and cerebellum, as well as a reduction in gray matter within other brain structures such as the amygdala, hippocampus, putamen, caudate, thalamus, and pallidum [13]. Additionally, functional magnetic resonance imaging (fMRI) can be employed to study the developmental patterns of brain growth and identify alterations in neuronal networks following PAE [14]. Heart rate variability (HRV), which is based on fluctuations in time intervals between successive heartbeats, is another valuable tool in providing information about the autonomic nervous system activity [15]. Ethanol has been already proven, both in clinical studies and animal models, to cause deleterious effects on the fetal cardiovascular system, including smooth muscle and endothelial dysfunctions [16,17]. At the same time, both animal and human researches suggest that the association between PAE and the autonomic nervous system malfunction exists; however, the causal relationship and the nature of changes remain uncharacterized [18]. Thus, the main aim of our study was to investigate cardiovascular and autonomic nervous system abnormalities in male and female rat offsprings in response to PAE, including the analysis of sex-associated abnormalities.

## 2. Materials and Methods

### 2.1. Ethical Approval

This study was conducted following ethical, regulatory, and scientific principles with the approval and supervision of the local Animal Welfare Committee of Jagiellonian University (protocol number 705/2022).

### 2.2. Animals

Nine Wistar rats (6 females and 3 males, with a mean body weight of 160 g and 200 g, respectively) were used to conceive offspring. Upon arrival from the Jagiellonian University Medical College Animal House, animals were housed under controlled conditions—12 h light/12 h dark cycle, at a temperature of 22 ± 2 °C, and humidity of 55 ± 10%. Transparent cages had been placed near each other to allow visual, auditory, and olfactory interaction. Each cage had been equipped with appropriate bedding products and habitat enrichment. Tap water and standard dry chow were available ad libitum: protein 25%, fat 8%, carbohydrates 67%, and metabolizable energy 2.86 kcal/g (Labofeed B, Kcynia, Poland).

### 2.3. Model Protocol

**Figure 1:**
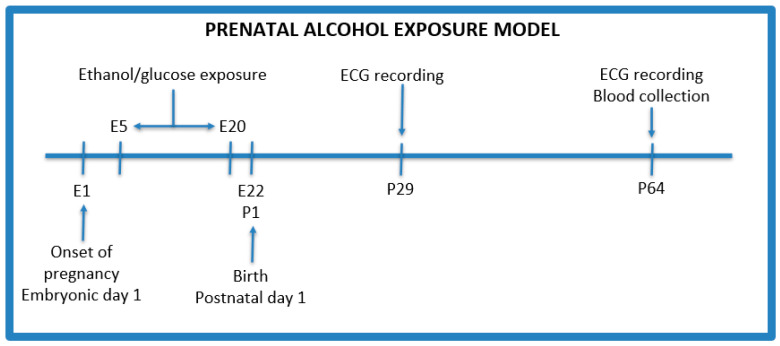
Prenatal alcohol exposure model. E—embryonic day, P—postnatal day.

The PAE protocol was adapted from Thomas et al. with modifications (Figure 1) [19]. After an initial acclimation period, male rats were confined with female rats for mating for 48 h. Males were used only as breeders in a ratio of 1 male to 2 females per cage, and were removed from the cages after the confirmation of pregnancy. Following the process of conception (one female did not become pregnant), 5 female rats were weighed and divided into the following groups:

1. Control group (n = 2)—isocaloric glucose solution 40% (*w*/*v*) given by oral gavage administered every day between 5 and 20 gestational days;
2. PAE group (n = 3)—3 g/kg b.w ethanol given by oral gavage administered every day between 5 and 20 gestational days with the following concentrations: 28.5% (*v*/*v*) ethanol from gestational day 5 to 17, and on the 18th, 19th, and 20th day, the dose of ethanol was reduced to 75%, 50%, and 25%, respectively, of the maximum dose to avoid withdrawal symptoms after the birth of the offspring.

Additionally, on the 8th, 11th, 15th, and 18th day of pregnancy, female rats received 8 g/kg b.w. of thiamine intramuscularly to prevent its deficiency after alcohol administration [20].

After 22 days of pregnancy, rats gave birth to 26 pups in total—10 (female: 5, male: 5) from the control group and 16 (female: 8, male: 8) from the PAE group. Offsprings stayed with their mothers during the feeding period, i.e., 21 days after birth. Then, they were isolated from their mother and divided into groups of 4–5 individuals to minimize the distress of isolation. Mothers were euthanized after weaning.

### 2.4. Electrocardiographic Recordings

The electrocardiographic (ECG) recordings were conducted by 2 independent researchers and took place on the 29th and 64th day of life of the newborn rats (examples of raw electrocardiographic data are provided in Supplementary Materials, Figure S1). Before each measurement, rats were anesthetized by intraperitoneal injection of ketamine/xylazine. Then, the chest and joints were gently shaved to facilitate electrode sticking, provide better signal quality, and narrow the incidence of artifacts. ECG measurements were carried out using LabChart 5.4.2 Pro (ADInstruments, Sydney, Australia). The animals were kept supine during anesthesia and testing, and at a constant body temperature provided by a heating pad with a rat temperature sensor (Physitemp, New York, NY, USA). The following linear and non-linear heart rate variability (HRV) parameters were measured as previously described: heart rate (HR), the standard deviation of NN intervals (SDNN), the root mean square of successive differences between normal heartbeats (RMSSD), low-frequency band (LF), high-frequency band (HF), the low-frequency-band-to-high-frequency-band ratio (LF/HF), the standard deviation of the first measurement (SD1), the standard deviation of the second measurement (SD2), the SD2-to-SD1 ratio (SD1/SD2), the approximate entropy (ApEn), the sample entropy (SampEn), the detrended fluctuation analysis short-term frequency (DFA_α1_), and the detrended fluctuation analysis long-term frequency (DFA_α2_). After the end of the procedure, the rats were moved into individual cages to recover from anesthesia before being placed into the standard housing environment.

SDNN is considered to be an indicator of overall heart rate variability or sympathetic tone, while RMSSD is mainly affected by vagal nerve neural activity [21,22,23]. LF correlates with sympathetic neural activity, HF correlates with parasympathetic nervous activity, and the LF/HF serves as an indicator of sympathovagal balance [23]. SD1 is affected by parasympathetic nervous activity, SD2 is affected by sympathetic nervous activity, and SD2/SD1 represents sympathovagal balance [24]. ApEn and SampEn are metrics that measure the entropy of heart rate, typically linked to a more intricate rhythm [25]. DFA1 is associated with the LF/HF ratio, reflecting the sympathovagal ratio, while DFA2 is associated with LF, suggesting sympathetic activity [26,27].

### 2.5. Biochemical Analysis

Blood samples from the jugular vessels were collected on the 68th day of life of the newborn rats in plastic tubes immediately after decapitation and incubated for at least 30 min at 4 °C to induce clot formation. After centrifugation at 1500× *g* for 20 min at 4 °C (Megafuge 1.0R, Heraeus Instruments, Hanau, Germany), serum samples were separated and kept frozen at −20 °C until further analysis. All measurements were performed in duplicate. Aspartate aminotransferase (AST), alanine aminotransferase (ALT), sodium (Na), potassium (K), magnesium (Mg), creatinine (Creat), cholesterol (Chol), high-density lipoprotein (HDL), non-high-density lipoprotein (Non-HDL), low-density lipoprotein (LDL), and triglyceride (TG) levels were determined in the blood serum using photometric assays measured with Roche/Hitachi Cobas c 701 and Roche/Hitachi Cobas c 501 analyzers.

### 2.6. Statistical Analysis

Results were presented as the mean with standard error (SE). Two-way repeated ANOVA was performed, with ethanol exposition and sex as factors, followed by a Bonferroni post hoc test. Differences were considered statistically significant when *p* < 0.05. The statistical analysis was conducted using the Statistica software (version 13, TIBCO Software).

## 3. Results

PAE was associated with elevated levels of ALT and AST, particularly in male rats (*p* = 0.0350 and *p* = 0.8708, respectively) (Table 1 and Table 2, Figure S2). Furthermore, PAE resulted in a notable elevation in creatinine concentration, with a more pronounced effect observed in female rats (*p* = 0.0079 and 0.0081, respectively) (Figures S3 and S4). Electrolyte homeostasis, specifically sodium and magnesium concentration, was not shown to be affected by PAE. Although differences in potassium levels between the sexes were noted in both experimental groups, these were not statistically significant (*p* = 0.5321). PAE affected lipid metabolism (cholesterol, high-density lipoprotein, non-high-density lipoprotein, low-density lipoprotein, and triglycerides), although no statistically significant changes were observed. The sex influence on potassium, cholesterol, non-HDL, and LDL concentration were observed in both the control and PAE groups, except for HDL concentration in the PAE group.

RMSSD exhibited higher values in the PAE group, particularly in female offsprings on the 29th day of the experiment (*p* = 0.0092) (Figure S5). Furthermore, there was a shift towards the parasympathetic activity observed in the PAE group based on SD1, and such alterations were particularly detected in the first assessment (*p* = 0.0093) (Figure S6). The mean heart rate (HR) on the 29th day was decreased in the PAE group in comparison to the control group (Table 3), yet with diminished disparities on the 64th day (*p* = 0.0951) (Figure S7). Additionally, the mean heart rate on the 29th day tended to be slightly different between female and male PAE offsprings (*p* = 0.1530). What is more, the mean HR tended to be lower in female PAE offsprings in comparison to the control female rats on the 29th day, but this disparity diminished by day 64 (Table 4). An increase in SDNN values was observed in the PAE group on the 29th day (Table 3), and early SDNN disparities were noted to a greater extent in female offsprings, but these changes were not statistically significant (*p* = 0.0638 and *p* = 0.3482, respectively) (Figure S8). Furthermore, a modest trend toward reduction in the heart rate entropy was noted in the PAE group but with no significant changes in relation to sex (Table 4). Finally, PAE does not appear to have significant effects on the weight of animals (Figure S9).

## 4. Discussion

HRV is a measure of complex neurocardiac function resulting from the interplay between the heart and brain, particularly governed by the autonomic nervous system (ANS). Furthermore, evaluating HRV provides indirect insight into ANS function, but also HRV characteristics can serve as noninvasive markers of cardiovascular well-being.

A decrease in heart rate following PAE protocol may have resulted from impaired autonomic nervous system development. Initially, after birth, heart rate is primarily controlled by the sympathetic nervous system (SNS) due to its early development. Throughout the maturation of the autonomic nervous system, the vagal impact on the heart increasingly augments. However, PAE has been found to intensify parasympathetic tone and potentially induce bradycardia [28]. The observed decrease in the mean HR could reflect such parasympathetic dominance. The increase in SDNN may be attributed to the activity of both the parasympathetic and sympathetic nervous systems [29]. The observed increase in SDNN does not seem to be linked to increased sympathetic activity in the PAE group. Numerous prior research has established a relationship between PAE and an increased risk of congenital cardiac abnormalities, such as the development of a thinner interventricular septum [30,31]. Additionally, it has been observed that PAE impacts the formation of endocardial cushions, leading to a greatly heightened susceptibility to atrioventricular canal defects. Eventually, PAE has an impact on the atrioventricular valve, resulting in the thinning of the cusps [32]. It is noteworthy to emphasize that even a single prenatal exposure to ethanol has the potential to result in significant congenital cardiac abnormalities [30]. What is more, Ling et al. suggested a link between PAE and anomalies in the heart’s conduction system, with a particular focus on the atrioventricular junction. The atrioventricular junction serves a crucial role in facilitating conduction delay, which is essential for ensuring optimal cardiac function [30].

The cardiovascular function of the female offspring population seems to be more noticeably affected by PAE. It should be noted that such observations were made regardless of the applied PAE protocol [33,34,35]. The PAE model as described by Thomas et al. was used with modifications [19]. According to the research conducted by Teraseki et al. and Collier et al., the dose of ethanol was reduced to 2.5 mg/kg b.w [34,35]. The dosage mentioned is considered appropriate for cases of moderate exposure. Due to a moderate dose of ethanol, it was feasible to reduce the frequency of oral gavage administration from twice daily to once daily, decreasing the distress experienced by animals. Furthermore, the administration of thiamine via intramuscular injection was introduced to prevent thiamine deficiency [20]. Similarly, Burgess et al. and Walton et al. have already documented notable distinctions in cardiovascular system performance and cardiovascular responsiveness among male and female children [36,37].

A notable elevation in alanine aminotransferase levels and a modest rise in asparagine aminotransferase levels were noted in the PAE group, irrespective of sex. Prior research also demonstrated such elevations due to PAE [38]. This phenomenon could be attributed to both the direct toxic impact on liver tissue and consequential liver dysfunction with impaired protein synthesis and glutathione activity, as well as the dysregulation of protein metabolism, namely alterations in enzyme phosphorylation and glycosylation [39,40]. Furthermore, PAE induced changes in glucose metabolism, such as increased gluconeogenesis, gluconeogenic genes, and oxidative and endoplasmic reticulum stresses [41]. Addolorato et al. reported a reduction in liver glutathione and ATP levels in the fetal population from mothers exposed to ethanol during pregnancy. Additionally, the authors observed the presence of mild-to-moderate intracellular steatosis [42]. Furthermore, the increase in creatinine levels can be attributed to the multifactorial effects of prenatal ethanol consumption. A correlation between PAE and several renal complications, including ureteral obstruction, renal hypoplasia, and hydronephrosis, as well as its effects on vascular function, leading to artery stiffness through the alteration of endothelial and smooth muscle function were reported [17,43,44]. Prior research also demonstrated a nephron deficit with increased apoptosis and an elevation in blood pressure [43,45]. The observed reduction in potassium levels in PAE rats could be either attributed to these renal problems or a dysfunctional hypothalamus–pituitary–adrenal axis, a phenomenon that has been observed both in human and animal participants, or abnormalities in the renin–angiotensin–aldosterone system [36,46,47]. What is more, Hu et al. reported an elevated concentration in cholesterol in adult rats that were subjected to ethanol throughout pregnancy, which aligns with the findings of our research, and abnormalities in the hypothalamus–pituitary–adrenal axis were reported to have an impact on lipid metabolism [48,49].

In conclusion, PAE led to a shift in autonomic nervous system function towards parasympathetic activity. These changes were particularly more significant in the first assessment. Additionally, significant discrepancies in creatinine and ALT levels were observed, indicating a complex pathway of the adverse effects of PAE. Although the long-term effects remain uncertain and difficult to predict, our results are relevant for further research into the metabolic and neurogenic consequences of prenatal alcohol exposure. The use of HRV methods could potentially aid in identifying disorders with sparse manifestations, given the systemic effects that present an extremely heterogeneous picture of the disease.

## Figures and Tables

**Table 1:** Biochemical parameters. Data are expressed as mean ± standard error (SE). Groups: control rats received glucose p.o. during pregnancy; PAE rats received ethanol p.o. during pregnancy. * demonstrates statistically significant differences (*p* < 0.05) in the PAE group compared to the control group.

	Control	PAE	*p* Value
AST [IU]	150.38 ± 6.49	151.87 ± 5.62	NS
ALT [IU]	46.88 ± 1.57	51.20 ± 1.12 *	0.0350
Na [mmol/L]	144.00 ± 0.42	144.87 ± 0.34	NS
K [mmol/L]	5.6 ± 0.13	5.9 ± 0.11	NS
Mg [mmol/L]	1.01 ± 0.02	0.99 ± 0.01	NS
Creat [mmol/L]	29.99 ± 1.02	33.47 ± 0.67 *	0.0079
Chol [mmol/L]	1.56 ± 0.07	1.70 ± 0.04	NS
HDL [mmol/L]	1.00 ± 0.05	1.07 ± 0.03	NS
Non-HDL [mmol/L]	0.57 ± 0.02	0.62 ± 0.02	NS
LDL [mmol/L]	0.18 ± 0.02	0.21 ± 0.1	NS
TG [mmol/L]	1.66 ± 0.10	1.52 ± 0.07	NS

NS—not significant.

**Table 2:** Biochemical parameters concerning sex. Data are expressed as mean ± standard error (SE). Groups: control rats received glucose p.o. during pregnancy; PAE rats received ethanol p.o. during pregnancy. * demonstrates statistically significant differences (*p* < 0.05) in the PAE group compared to the control.

	Control		PAE		
	F	M	F	M	*p* Value
AST [IU]	151.80 ± 21.28	148.00 ± 16.09	142.75 ± 16.10	162.29 ± 23.80	NS
ALT [IU]	46.60 ± 5.86	47.33 ± 0.58	50.63 ± 3.58	51.86 ± 5.30	NS
Na [mmol/L]	144.00 ± 1.41	144.00 ± 1.00	144.50 ± 0.93	145.29 ± 1.60	NS
K [mmol/L]	5.30 ± 0.11	5.99 ± 0.22	5.24 ± 0.24	5.77 ± 0.39	NS
Mg [mmol/L]	0.98 ± 0.05	1.05 ± 0.03	0.99 ± 0.04	0.99 ± 0.05	NS
Creat [mmol/L]	28.92 ± 2.83	31.77 ± 2.37	34.39 ± 0.97 *	32.41 ± 3.51	0.0081
Chol [mmol/L]	1.44 ± 0.11	1.77 ± 0.12	1.63 ± 0.17	1.79 ± 0.07	NS
HDL [mmol/L]	0.91 ± 0.10	1.14 ± 0.06	1.05 ± 0.14	1.10 ± 0.06	NS
Non-HDL [mmol/L]	0.52 ± 0.02	0.65 ± 0.03	0.57 ± 0.06	0.67 ± 0.06	NS
LDL [mmol/L]	0.13 ± 0.01	0.26 ± 0.00	0.17 ± 0.04	0.26 ± 0.03	NS
TG [mmol/L]	1.78 ± 0.26	1.46 ± 0.26	1.45 ± 0.27	1.60 ± 0.31	NS

NS—not significant.

**Table 3:** Time-dependent changes in physical and heart rate variability parameters. Data are expressed as mean ± standard error (SE). Groups: control rats received glucose p.o. during pregnancy; PAE rats received ethanol p.o. during pregnancy. * demonstrates statistically significant differences (*p* < 0.05) in the PAE group compared to the control group.

	Day 29		Day 64		
	Control	PAE	Control	PAE	*p* Value
Weight [g]	76.5 ± 1.89	79.06 ± 1.70	221.22 ± 19.92	234.93 ± 14.62	NS
Mean HR [beats/min]	388.57 ± 16.28	343.55 ± 17.52	308.24 ± 10.32	300.69 ± 14.62	NS
SDNN [ms]	1.84 ± 0.53	3.69 ± 0.91	2.12 ± 0.15	2.11 ± 0.33	NS
RMSSD [ms]	2.05 ± 0.27	5.21 ± 1.14 *	2.87 ± 0.31	3.01 ± 0.40	0.0092
LF n.u.	10.88 ± 2.81	5.72 ± 1.00	17.32 ± 6.64	12.48 ± 3.32	NS
HF n.u.	89.11 ± 2.81	94.27 ± 1.00	82.68 ± 6.64	87.52 ± 3.32	NS
LF/HF	0.13 ± 0.04	0.06 ± 0.01	0.31 ± 0.18	0.17 ± 0.06	NS
SD1 [ms]	1.45 ± 0.19	3.69 ± 0.81 *	2.03 ± 0.22	2.13 ± 0.28	0.0093
SD2 [ms]	2.04 ± 0.77	3.63 ± 1.01	2.11 ± 0.23	2.05 ± 0.39	NS
SD2/SD1	1.30 ± 0.28	0.97 ± 0.07	1.21 ± 0.31	0.94 ± 0.06	NS
ApEn	1.01 ± 0.05	0.99 ± 0.05	1.13 ± 0.02	1.12 ± 0.02	NS
SampEn	1.31 ± 0.13	1.17 ± 0.08	1.65 ± 0.12	1.55 ± 0.07	NS
DFA1	0.36 ± 0.05	0.24 ± 0.02	0.43 ± 0.11	0.35 ± 0.05	NS
DFA2	0.59 ± 0.11	0.45 ± 0.08	0.71 ± 0.11	0.65 ± 0.04	NS

NS—not significant.

**Table 4:** Time-dependent changes in physical and heart rate variability parameters concerning sex. Data are expressed as mean ± standard error (SE). Groups: control rats received glucose p.o. during pregnancy; PAE rats received ethanol p.o. during pregnancy.

	Day 29				Day 64			
	Control		PAE		Control		PAE	
	F	M	F	M	F	M	F	M
Weight [g]	71.6 ± 1.03	81.40 ± 1.72	74.25 ± 1.56	83.88 ± 1.82	171.20 ± 2.96	283.75 ± 4.44	183.29 ± 2.15	286.57 ± 5.76
Mean HR [beats/min]	381.37 ± 25.7	397.57 ± 21.0	301.21 ± 13.2	385.90 ± 21.4	301.57 ± 14.3	319.35 ± 14.6	296.33 ± 10.0	304.43 ± 7.8
SDNN [ms]	2.30 ± 0.94	1.26 ± 0.17	4.88 ± 1.70	2.49 ± 0.40	2.01 ± 0.18	2.30 ± 0.25	1.89 ± 0.33	2.29 ± 0.57
RMSSD [ms]	2.31 ± 0.41	1.72 ± 0.32	6.77 ± 2.08	3.64 ± 0.66	2.95 ± 0.34	2.74 ± 0.72	2.65 ± 0.49	3.33 ± 0.62
LF n.u.	9.95 ± 4.74	12.06 ± 3.00	5.04 ± 1.48	6.40 ± 1.41	12.76 ± 3.85	24.92 ± 17.77	11.59 ± 4.68	13.25 ± 5.02
HF n.u.	90.05 ± 4.74	87.94 ± 3.00	94.96 ± 1.48	93.58 ± 1.41	87.24 ± 3.85	75.08 ± 17.77	88.41 ± 4.68	86.75 ± 5.02
LF/HF	0.12 ± 0.07	0.14 ± 0.04	0.05 ± 0.02	0.07 ± 0.02	0.16 ± 0.05	0.56 ± 0.48	0.15 ± 0.08	0.18 ± 0.09
SD1 [ms]	1.63 ± 0.29	1.22 ± 0.23	4.80 ± 1.47	2.58 ± 0.46	2.09 ± 0.24	1.94 ± 0.51	1.88 ± 0.34	2.36 ± 0.44
SD2 [ms]	2.64 ± 1.38	1.30 ± 0.11	4.90 ± 1.93	2.35 ± 0.37	1.90 ± 0.18	2.45 ± 0.53	1.89 ± 0.34	2.18 ± 0.69
SD2/ SD1	1.45 ± 0.52	1.12 ± 0.10	0.97 ± 0.10	0.97 ± 0.10	0.95 ± 0.12	1.64 ± 0.82	1.04 ± 0.08	0.85 ± 0.08
ApEn	0.96 ± 0.06	1.06 ± 0.08	1.00 ± 0.06	0.98 ± 0.08	1.16 ± 0.03	1.09 ± 0.02	1.14 ± 0.04	1.11 ± 0.03
SampEn	1.31 ± 0.22	1.31 ± 0.14	1.19 ± 0.10	1.16 ± 0.13	1.76 ± 0.14	1.45 ± 0.18	1.67 ± 0.09	1.46 ± 0.09
DFA1	0.36 ± 0.09	0.35 ± 0.05	0.23 ± 0.04	0.26 ± 0.03	0.36 ± 0.06	0.54 ± 0.30	0.38 ± 0.07	0.32 ± 0.07
DFA2	0.63 ± 0.19	0.54 ± 0.06	0.44 ± 0.10	0.45 ± 0.13	0.66 ± 0.14	0.78 ± 0.19	0.75 ± 0.07	0.57 ± 0.03

## Data Availability

The study data will be accessible from the corresponding author following a reasonable request.

## Supplementary Materials

The following supporting information can be downloaded at https://www.mdpi.com/article/10.3390/pediatric16020024/s1, Figure S1: Examples of raw electrocardiographic data.; Figure S2: ALT, concerning group; Figure S3: Creatinine, concerning group; Figure S4: Creatinine, concerning group and sex; Figure S5: RMSSD, concerning day and group; Figure S6: SD1, concerning day and group; Figure S7: Heart rate, concerning day and group; Figure S8: SDNN, concerning day and group; Figure S9: Weight, concerning day and group.
