# Supplementary material for: The Impact of Prenatal Alcohol Exposure on the Autonomic Nervous System and Cardiovascular System in Rats in a Sex-Specific Manner
Source: Pediatr Rep. 2024 Apr 9;16(2):278–87. doi: 10.3390/pediatric16020024 (PMC11036276; doi:10.3390/pediatric16020024)
Supplement: Supplementary file 1 [file pediatrrep-16-00024-s001.zip › pediatrrep-2796540-supplementary.pdf]

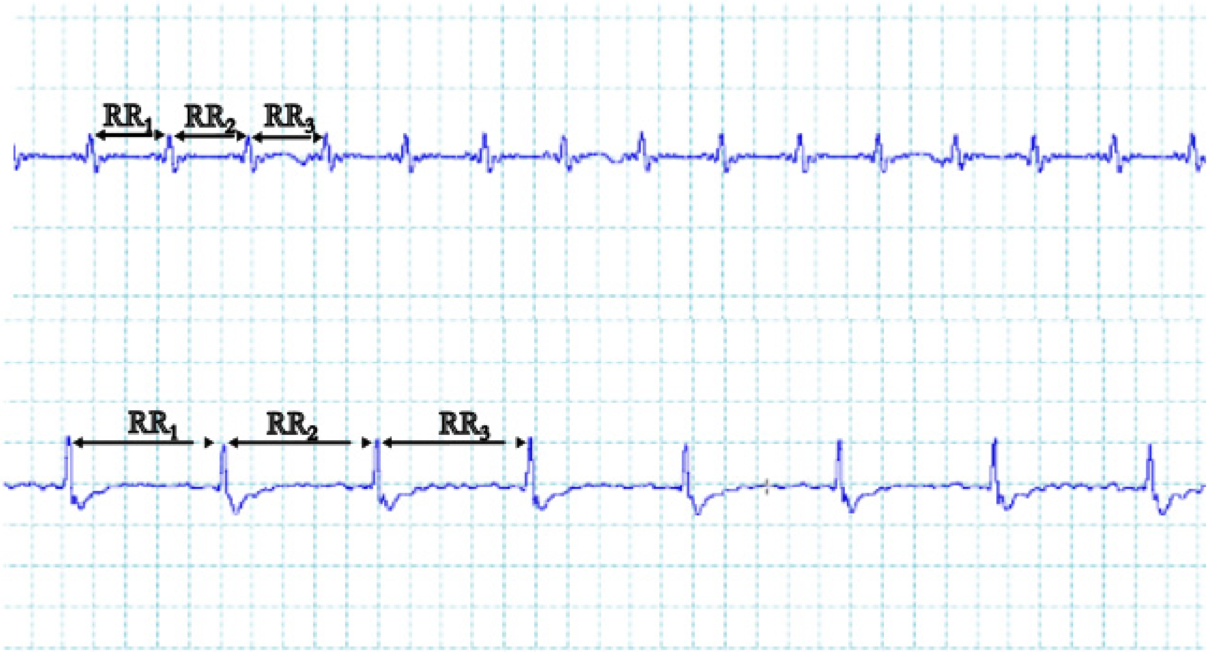

Figure S1. Examples of raw electrocardiographic data. Subsequent RR intervals are indicated.

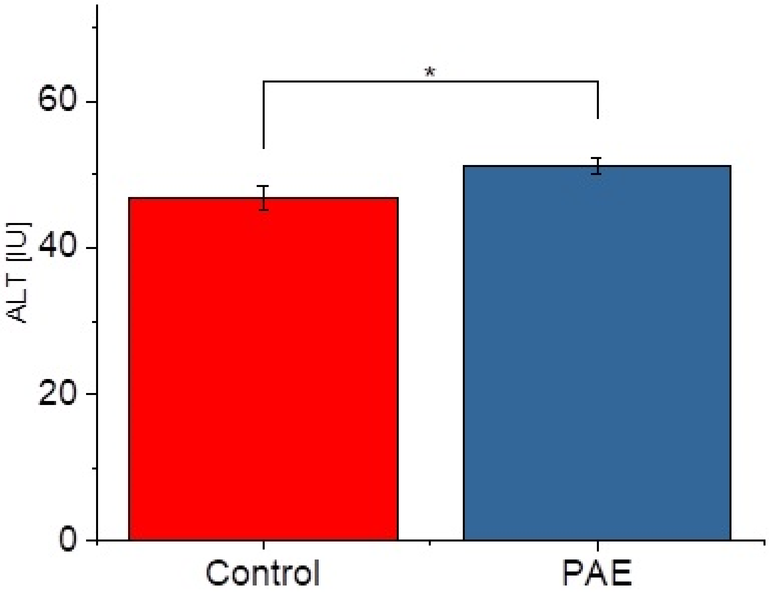

Figure S2. ALT, concerning group. Data are expressed as mean ± standard error (SE). Groups: control rats received glucose p.o. during pregnancy; PAE rats received ethanol p.o. during pregnancy. * demonstrates a statistically significant difference ($p<0.05$)

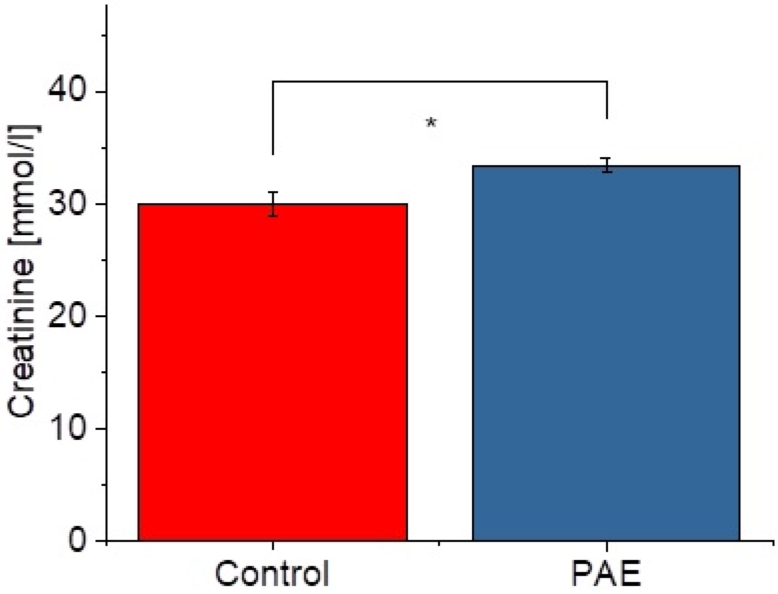

Figure S3. Creatinine, concerning group. Data are expressed as mean ± standard error (SE). Groups: control rats received glucose p.o. during pregnancy; PAE rats received ethanol p.o. during pregnancy. * demonstrates a statistically significant difference ($p<0.05$)

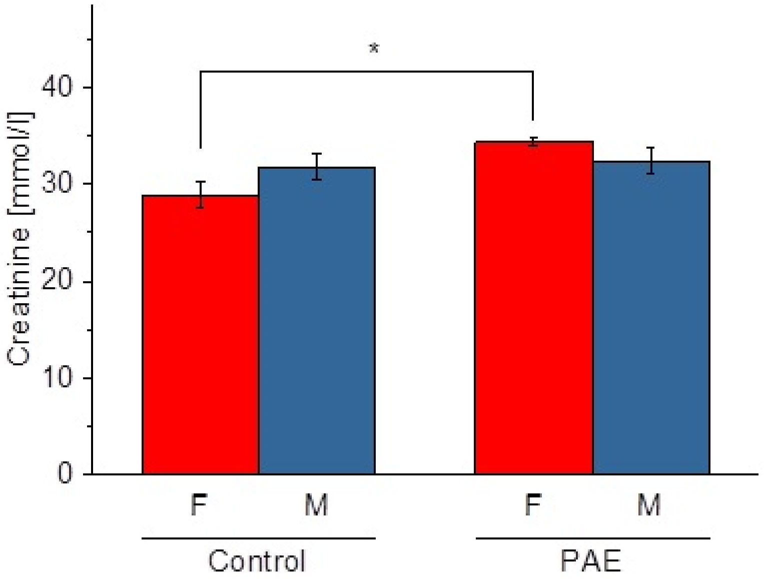

Figure S4 Creatinine, concerning group and sex. Data are expressed as mean ± standard error (SE). Groups: control rats received glucose p.o. during pregnancy; PAE rats received ethanol p.o. during pregnancy; sex: F-female, M- male. * demonstrates statistically significant differences ($p<0.05$)

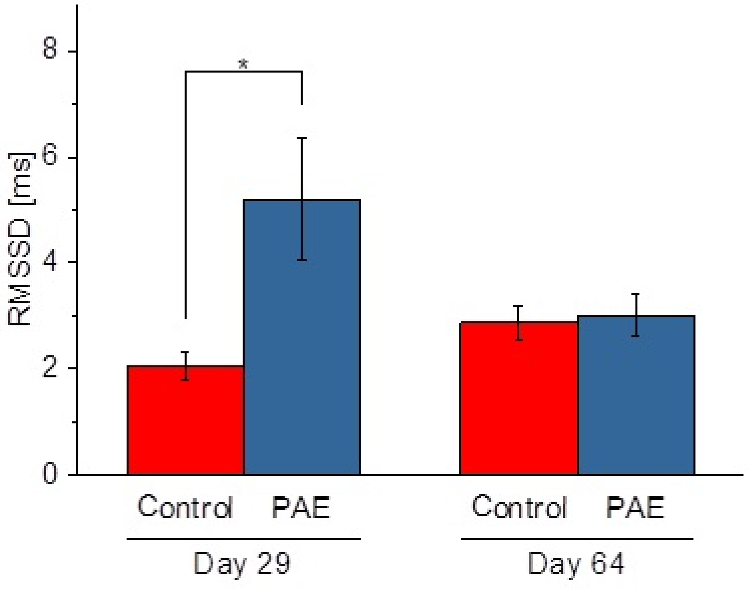

Figure S5. RMSSD, concerning day and group. Data are expressed as mean ± standard error (SE). Groups: control rats received glucose p.o. during pregnancy; PAE rats received ethanol p.o. during pregnancy. * demonstrates statistically significant differences ($p<0.05$)

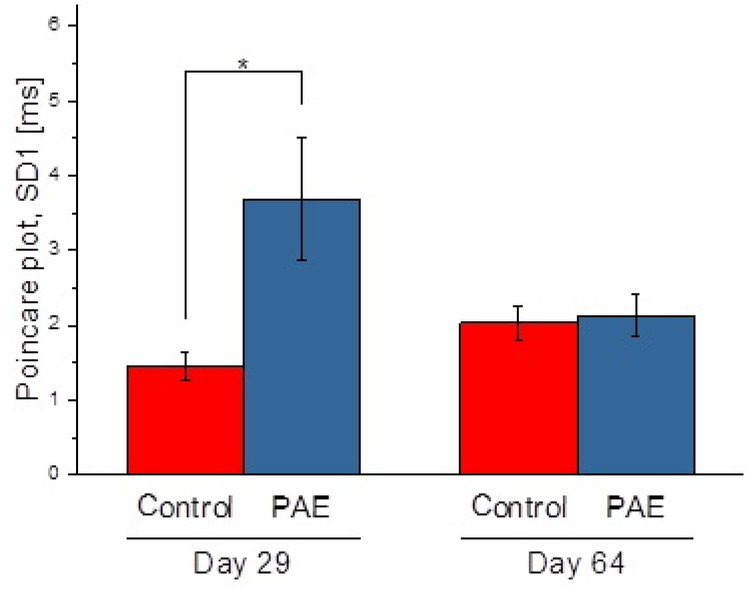

Figure S6. SD1, concerning day and group. Data are expressed as mean ± standard error (SE). Groups: control rats received glucose p.o. during pregnancy; PAE rats received ethanol p.o. during pregnancy. * demonstrates statistically significant differences ($p<0.05$)

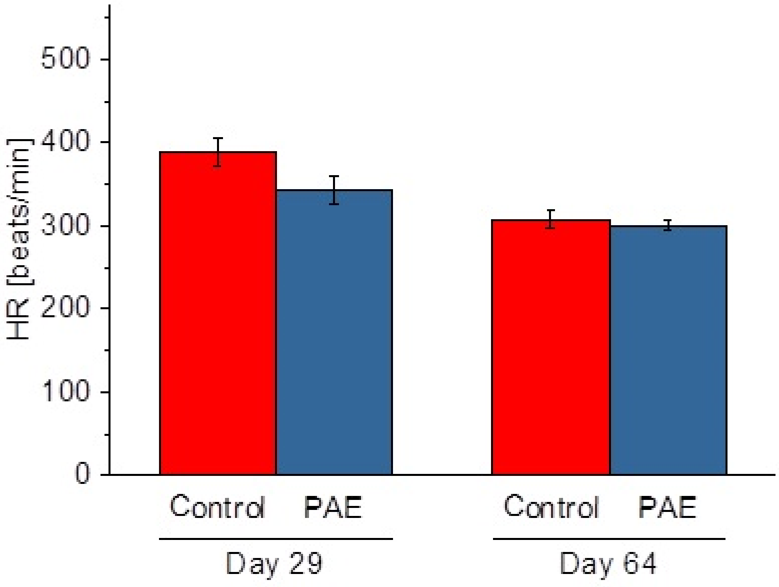

Figure S7. Heart rate, concerning day and group. Data are expressed as mean ± standard error (SE). Groups: control rats received glucose p.o. during pregnancy; PAE rats received ethanol p.o. during pregnancy. * demonstrates statistically significant differences ($p<0.05$)

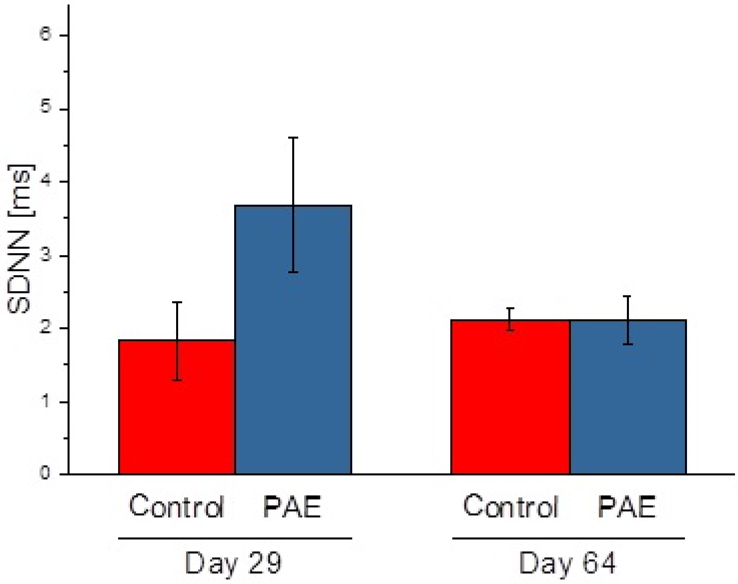

Figure S8. SDNN, concerning day and group. Data are expressed as mean ± standard error (SE). Groups: control rats received glucose p.o. during pregnancy; PAE rats received ethanol p.o. during pregnancy. * demonstrates statistically significant differences ($p<0.05$)

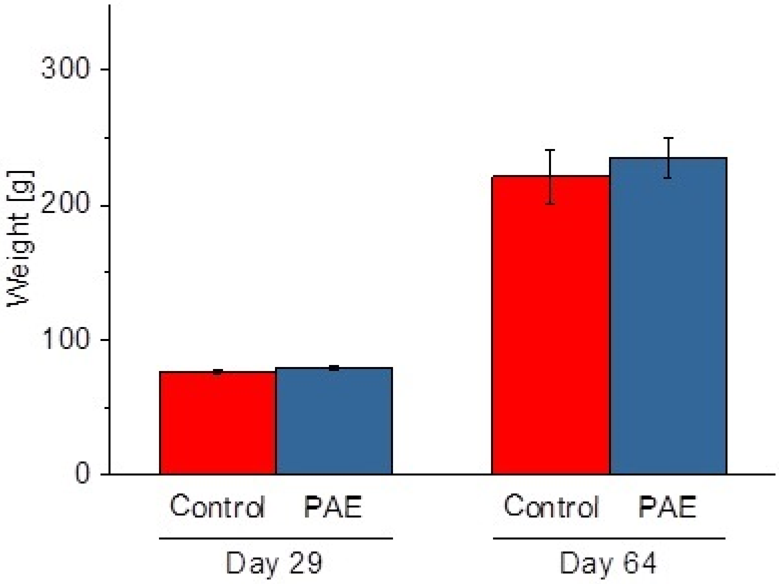

Figure S9. Weight, concerning day and group. Data are expressed as mean ± standard error (SE). Groups: control rats received glucose p.o. during pregnancy; PAE rats received ethanol p.o. during pregnancy. * demonstrates statistically significant differences ($p<0.05$)
